# Development and validation of a multivariable risk factor questionnaire to detect oesophageal cancer in 2-week wait patients^☆^

**DOI:** 10.1016/j.clinre.2023.102087

**Published:** 2023-03

**Authors:** Kai Man Alexander Ho, Avi Rosenfeld, Áine Hogan, Hazel McBain, Margaret Duku, Paul BD Wolfson, Ashley Wilson, Sharon MY Cheung, Laura Hennelly, Lester Macabodbod, David G Graham, Vinay Sehgal, Amitava Banerjee, Laurence B Lovat, SPIT Study Group Collaborators, Olivia Adu-Anti, Kalliopi Alexandropoulou, Ameena Ayub, Nicky Barnes, Peter Basford, Ellen Brown, Jeffrey Butterworth, Heather Button, Ellie Clarke, Alexandra Cope, Jessica Cordle, Joana Da Rocha, John DeCaestecker, Anjan Dhar, Jason Dunn, Martin Ebon, Stacey Forsey, Tracy Foster, Edith Gallagher, Helen Graham, Fiona Gregg, Philip Hall, Sandra Jackson, Nicole Kader, Sudarshan Kadri, Sandhya Kalsi, Richard Keld, Chun Lee, Hui Yann Lee, Andy CY Li, Gideon Lipman, Inder Mainie, Julie Matthews, Cheryl Mendonca, Danielle Morris, Vinod Patel, Philip Paterson, Rosemary Phillips, Elizabeth Ratcliffe, Cait Rees, Joana Da Rocha, Radu Rusu, Heather Savill, Sharan Shetty, Leena Sinha, Bob Soin, Mamoon Solkar, Darmarajah Veeramootoo, Joanne Vere, Olivia Watchorn, Hendrik Wegstapel, Tracey White, Robert Willert, Susannah Woodrow, Sebastian Zeki

**Affiliations:** a St Mark's Hospital, London North West University Healthcare NHS Trust, Harrow, UK; b Royal Surrey County Hospital, Royal Surrey County Hospital NHS Foundation Trust, Guildford, UK; c Wexham Park Hospital, Frimley Health NHS Foundation Trust, Slough, UK; d St Richard's Hospital, Western Sussex Hospitals NHS Foundation Trust, Chichester, UK; e Darlington Memorial Hospital, County Durham and Darlington NHS Foundation Trust, Darlington, UK; f Royal Shrewsbury Hospital, Shrewsbury and Telford Hospital NHS Trust, Shrewsbury, UK; g Leicester General Hospital & Leicester Royal Infirmary, University Hospitals of Leicester NHS Trust, Leicester, UK; h St Thomas’ Hospital, Guy's and St Thomas’ NHS Foundation Trust, London, UK; i Lister Hospital, East and North Hertfordshire NHS Trust, Stevenage, UK; j Russells Hall Hospital, The Dudley Group NHS Foundation Trust, Dudley, UK; k Frimley Park Hospital, Frimley Health NHS Foundation Trust, Camberley, UK; l Belfast City Hospital, Belfast Health and Social Care Trust, Belfast, UK; m Royal Albert Edward Infirmary, Wrightington, Wigan and Leigh Teaching Hospitals NHS Foundation Trust, Wigan, UK; n Manchester Royal Infirmary, Manchester University NHS Foundation Trust, Manchester, UK; o Tameside General Hospital, Tameside and Glossop Integrated Care NHS Foundation Trust, Ashton-under-Lyne, UK; p Princess Alexandra Hospital, The Princess Alexandra Hospital NHS Trust, Harlow, UK; q Queen's Hospital, Barking, Havering and Redbridge University Hospitals NHS Trust, Romford, UK; r Medway Maritime Hospital, Medway NHS Foundation Trust, Gillingham, UK; a Division of Surgery and Interventional Science, University College London, Charles Bell House, 43-45 Foley Street, London W1W 7TY, UK; b Wellcome/EPSRC Centre for Interventional and Surgical Sciences (WEISS), University College London, Charles Bell House, 43-45 Foley Street, London W1W 7TY, UK; c Department of Computer Science, Jerusalem College of Technology, Havaad Haleumi 21, Givat Mordechai 91160 Jerusalem, Israel; d Department of Gastrointestinal Services, University College London Hospital, University College London Hospitals NHS Foundation Trust, 235 Euston Road, London NW1 2BU, UK; e Institute of Health Informatics, University College London, 222 Euston Road, London NW1 2DA, UK; f Department of Cardiology, St Bartholomew's Hospital, Barts Health NHS Trust, London EC1A 7BE, UK

**Keywords:** XX

## Abstract

**Introduction:**

Oesophageal cancer is associated with poor health outcomes. Upper GI (UGI) endoscopy is the gold standard for diagnosis but is associated with patient discomfort and low yield for cancer. We used a machine learning approach to create a model which predicted oesophageal cancer based on questionnaire responses.

**Methods:**

We used data from 2 separate prospective cross-sectional studies: the Saliva to Predict rIsk of disease using Transcriptomics and epigenetics (SPIT) study and predicting RIsk of diSease using detailed Questionnaires (RISQ) study. We recruited patients from National Health Service (NHS) suspected cancer pathways as well as patients with known cancer. We identified patient characteristics and questionnaire responses which were most associated with the development of oesophageal cancer. Using the SPIT dataset, we trained seven different machine learning models, selecting the best area under the receiver operator curve (AUC) to create our final model. We further applied a cost function to maximise cancer detection. We then independently validated the model using the RISQ dataset.

**Results:**

807 patients were included in model training and testing, split in a 70:30 ratio. 294 patients were included in model validation. The best model during training was regularised logistic regression using 17 features (median AUC: 0.81, interquartile range (IQR): 0.69–0.85). For testing and validation datasets, the model achieved an AUC of 0.71 (95% CI: 0.61–0.81) and 0.92 (95% CI: 0.88–0.96) respectively. At a set cut off, our model achieved a sensitivity of 97.6% and specificity of 59.1%. We additionally piloted the model in 12 patients with gastric cancer; 9/12 (75%) of patients were correctly classified.

**Conclusions:**

We have developed and validated a risk stratification tool using a questionnaire approach. This could aid prioritising patients at high risk of having oesophageal cancer for endoscopy. Our tool could help address endoscopic backlogs caused by the COVID-19 pandemic.

## Introduction

Oesophageal cancer represents the seventh most common cause of cancer morbidity and the sixth most common cause of cancer-related death worldwide [1]. There are two major histological subtypes: oesophageal squamous cell carcinoma (OSCC) and oesophageal adenocarcinoma (OAC). OSCC comprises 90% of oesophageal cancer cases worldwide and is predominantly found in Central Asia, East Asia and East Africa. Conversely, OAC make up the remainder but is the dominant histological subtype in the Western world, including the United Kingdom (UK) [2], [3], [4]. Crucially, oesophageal cancer is often diagnosed late; in the United Kingdom 48% of cases with available staging information are diagnosed at stage IV, while 10-year survival stands at 12%, significantly worse than other cancer types. [4,5] Although, gastro oesophageal junction (GOJ) cancers represent a heterogenous entity, they have common risk factors with oesophageal cancers. As such, historically they have been included in studies with oesophageal cancer [6].

While upper gastrointestinal (UGI) endoscopy remains the gold standard in the diagnosis of oesophageal and GOJ cancers, it is expensive, uncomfortable for the patient and has a low yield for cancer [7]. A UK series of over 580,000 patients demonstrated that only 2.1% of patients undergoing UGI endoscopy were subsequently found to have cancer, while other serious pathology such as peptic ulceration were found in a further 11.6% [7]. These figures suggest that better selection of patients who are likely to develop oesophageal cancer could help to prioritise higher risk patients, better manage demand for endoscopy and improve overall patient experience. Furthermore, previous work from our group demonstrated a nationwide endoscopic procedural backlog because of the COVID-19 pandemic [8]. In addition, during the first 6-months of the pandemic there were decreases in pathological diagnoses of Barrett's oesophagus and OAC [9]. There is potential for worse patient outcomes as a direct consequence of the disruption of endoscopy services, on top of the already poor patient outcomes for oesophageal cancer. Modelling studies have demonstrated that disruption to National Health Service (NHS) cancer pathways could lead to excess deaths and life years lost due to delays in diagnosis [10,11].

Several research groups have created scoring systems to try to improve detection of oesophageal cancer. The Edinburgh Dysphagia Scale (EDS) categorises patients into low or high risk for groups using a combination of both patient characteristics and symptoms. In a validation cohort it achieved a sensitivity of 98.4% but specificity was low at 9.3% [12,13].

Increasingly, machine learning (ML) methods, which apply mathematical approaches to generating computerised algorithms, have been used to develop triaging models, which could optimise use of resources. These models can calculate an individual's risk of having a disease [14]. Our group previously used an ML approach to develop a risk prediction model for Barrett's oesophagus with the aim of improving selection of patients who should be referred for UGI endoscopy for confirmation [14]. Similar approaches have been used in acute UGI bleeding for risk stratification and it even outperformed standard of care clinical scoring systems [15].

## Aims and objectives

The aim of this study was to use an ML approach to train, test and then independently validate a risk stratification tool which could be used to predict the risk of detecting oesophageal and GOJ cancers in unselected patients referred through NHS 2-week wait (2WW) suspected UGI cancer pathways. In addition, we aimed to trial the tool in a limited number of patients with gastric cancer, as both cohorts of patients require the same endoscopic investigation for diagnosis.

## Methods

### Participant selection and dataset description

Patients were selected from two separate prospective cross-sectional studies; the Saliva to Predict rIsk of disease using Transcriptomics and epigenetics (SPIT) study (ISRCTN: 11,921,553) and predicting RIsk of diSease using detailed Questionnaires (RISQ) study (ISRCTN: 74,930,639). For the SPIT study, participants were recruited from those referred for UGI endoscopy through the NHS 2WW suspected UGI cancer pathway from 19 UK hospitals between September 2017 and May 2022. Major inclusion criteria were age over 18 and ability to provide informed consent. Exclusion criterion was pregnancy. For the RISQ study, participants were recruited from 2 UK hospitals between January 2020 and May 2022 using identical inclusion and exclusion criteria. Patients in both studies completed a symptom and risk factor questionnaire either independently or with support from a research nurse immediately before undergoing diagnostic endoscopy. The SPIT study questionnaire consisted of 209 questions; it was multidimensional with different domains that are known to impact disease risk (Supplementary Table 1). Major symptoms for oesophageal cancer such as dysphagia, odynophagia and weight loss were included [16]. Questions on duration and severity of acid reflux symptoms were adapted from both the gastro oesophageal reflux disease (GORD) impact scale and GORD questionnaire [17], [18], [19]. We included wider questions on medication use, food intake, anxiety and depression, loneliness and local engagement as these have been found to affect health outcomes; we were interested to see if these factors may add additional richness to our model [20], [21], [22]. We used existing validated questionnaires in several sections of the questionnaire, including the Hospital Anxiety and Depression Score (HADS), the University of California Los Angeles (UCLA) 3 item loneliness scale and the dysphagia score by Mellow and Pinkas [23], [24], [25]. The RISQ questionnaire was a shortened version of the SPIT questionnaire with 17 questions (Supplementary Table 1), which were selected following initial model training [26]. Subsequent endoscopic and histological results were linked to the patient's questionnaire responses. As participants underwent endoscopic investigation as part of their routine care, endoscopists and histopathologists were both blinded to questionnaire responses. Questionnaires and outcomes were recorded using a bespoke electronic software programme (TrialSense, London, UK).

### Data handling

**Fig. 1:**
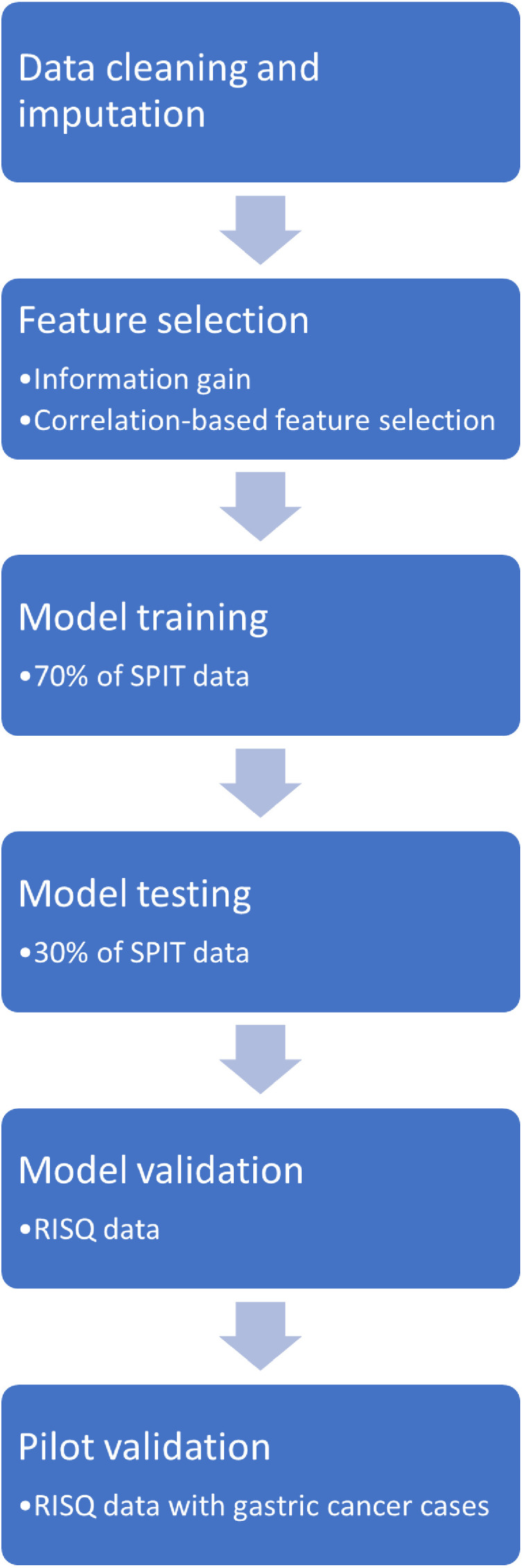
Process flow for model training, testing and validation.

All data handling and analysis was completed using R software version 4.1.2 (R Core Team, Vienna, Austria) [27]. Prior to data analysis the dataset was manually cleaned for data input issues and errors. We excluded any fields which had greater than 20% of responses missing. We performed missing data imputation using the ‘missForest’ package [28]. The process flow for our study is shown in Fig. 1.

### Feature selection

We used feature analysis to determine the most important predictors for oesophageal cancer. We used both information gain and chi-squared correlation-based feature selection from the ‘FSelector’ package, as per our previous methodology, with a 50:50 weighting given to each feature before producing a final ranking. [14,29]. In brief, information gain is an ML method where each feature is compared separately as to its correlation with the variable of interest. Correlation based feature selection assesses multiple features and removes any features which are high correlated with each other [14]. We were then able to select the most discriminating features to predict the presence of oesophageal cancer.

### Model training and testing

We split the SPIT dataset in a 70:30 ratio for model training and testing respectively. We performed 10-fold cross-validation during model training. We used seven supervised ML methods from the R software ‘caret’ package: [30]. linear discriminant analysis (caret function: lda), classification and regression tree (cart), k-nearest neighbour (knn), support vector machines (svm), random forest (rf), logistic regression (glm) and regularised logistic regression (glmnet). In particular, regularised logistic regression applies either ridge or lasso regularisation, which help to simplify any generated models and prevent overfitting of data [31]. This is denoted by an elastic net mixing parameter termed alpha [34]. A regularisation coefficient (lambda) is applied to the overall model which also penalises more complex models [31]. We assessed the performance of the model using receiver operating characteristic curves (ROC) and calculated the area under the ROC curve (AUC) using the ‘pROC’ package [32]. Our method automatically selects the optimal regularisation and lambda value to maximise AUC. Finally, to ensure that the model was weighted such that there was an increased penalty for misclassification of cancers, we applied a cost function to our final model using the ‘ROCR’ package [33]. The penalty for false negatives (i.e., missing a cancer case) was set at 50 times greater than false positives. This was then used to determine the ideal threshold above which the model would predict the presence of cancer. We assessed the performance of the cost function and the associated threshold using sensitivity, specificity, positive predictive value (PPV) and negative predictive value (NPV).

### Model validation

Patients in the validation dataset were from the RISQ study. We additionally enriched this cohort with further patients with confirmed oesophageal cancer. We also collected data from patients with confirmed gastric cancer to assess the performance of the model in this group.

### Ethical approval

Ethical approval was obtained for both the SPIT Study (Coventry and Warwickshire Research Ethics Committee: 17/WM/0079) and the RISQ Study (South Central - Oxford B Research Ethics Committee: 19/SC/0382).

### Statistical analysis

For risk prediction models, there is no generally accepted approach to estimate sample size requirements for derivation and validation of risk prediction models. Discrete variables are presented as numbers and percentages, while continuous variables are presented as mean and standard deviation (SD) or median and inter-quartile range (IQR). To compare between normal and cancer groups, we used t-tests or chi-squared (Χ^2^) tests depending on the variable. A p-value of ≤0.05 was taken as significant. We followed TRIPOD guidelines in the preparation of this manuscript [34].

## Results

### Demographics

Table 1

**Table 1:** Demographic and questionnaire Responses for included patients in training, testing and validation data sets of top ranked features. Numbers in brackets following feature (e.g. no, never (0)) denote coding used for model development. p-values are for chi-squared tests unless otherwise stated. FET= Fisher's Exact Test. KW=Kruskal Wallis Test.

	Training Data (*n* = 566)					Testing Data (*n* = 241)					Validation Data (*n* = 294)				
	Cancer Present	%	Cancer Absent	%	p value	Cancer Present	%	Cancer Absent	%	p value	Cancer Present	%	Cancer Absent	%	p value
**n**	65	11	501	89		27	11	214	89		42	14	252	86	
**Age**															
Mean	70.4		63.8		<0.001	70.6		63.5		0.013	70.8		52.1		<0.001
SD	10.1		13.3		KW	10.2		14		KW	9.96		17.1		
**Sex**															
Male (1)	45	69	208	42	<0.001	18	67	118	55	0.351	35	83	137	54	<0.001
Female (2)	20	31	293	58		9	33	96	45		7	17	115	46	
**Cancer Site**															
Oesophageal	52	80				25	93				35	83			
Gastro Oesophageal Junction	13	20				2	7				7	17			
**Cancer Histology**															
Adenocarcinoma	54	83				21	78				39	93			
Squamous Cell Carcinoma	5	8				3	11				3	7			
Other/Unknown	6	9				3	11				0	0			
**Ethnicity**															
White British/Irish/European	63	97	457	91	0.529	26	96	198	93	0.845	37	88	190	75	0.049
Mixed Race	1	2	1	0	FET	0	0	1	0	FET	0	0	5	2	FET
Asian	1	2	14	3		0	0	5	2		0	0	10	4	
Asian Other	0	0	8	2		0	0	1	0		4	10	13	5	
Black	0	0	11	2		1	4	5	2		1	2	12	5	
Other	0	0	10	2		0	0	4	2		0	0	22	9	
**Current/Previous Smoking**															
No (0)	26	40	265	53	0.061	10	37	101	47	0.416	10	24	137	54	<0.001
Yes (1)	39	60	233	47		17	63	112	52		32	76	115	46	
Unknown	0	0	3	1		0	0	1	0		0	0	0	0	
**Smoking Pack Years**															
Mean	13.4		6.8		0.029	12.8		6.5		0.078	14.3		7.1		0.013
SD	22.9		14.6		KW	20.4		11.4		KW	15.6		15.0		KW
Unknown	15		89			3		39			17		14		
**BMI**															
Mean	25.6		27.2		0.013	26.6		26.4		0.554	28.6		30.9		0.956
SD	4.5		6.0			7.5		5.2			8.5		10.2		
Unknown	2		19			2		4			38		223		
**Chest Pain Begin**															
No Chest Pain (0)	31	48	258	51	0.018	21	78	111	52	0.173	28	67	123	49	0.008
Less than 6 months (1)	20	31	69	14	FET	4	15	30	14	FET	7	17	13	5	FET
6 months to 1 year (2)	4	6	44	9		1	4	18	8		2	5	18	7	
1 to 5 years (3)	3	5	60	12		0	0	22	10		3	7	37	15	
5 to 10 years (4)	1	2	29	6		0	0	15	7		1	2	24	10	
10 to 20 years (5)	1	2	16	3		0	0	10	5		0	0	23	9	
More than 20 years (6)	1	2	16	3		0	0	5	2		1	2	14	6	
Unknown	4	6	9	2		1	4	3	1		0	0	0	0	
**Regurgitation Begin**															
No Regurgitation (0)	35	54	267	53	0.041	17	63	110	51	0.050	21	50	122	48	0.005
Less than 6 months (1)	15	23	54	11	FET	3	11	28	13	FET	9	21	11	4	FET
6 months to 1 year (2)	2	3	40	8		4	15	19	9		3	7	19	8	
1 to 5 years (3)	2	3	46	9		1	4	24	11		4	10	40	16	
5 to 10 years (4)	2	3	36	7		0	0	10	5		2	5	26	10	
10 to 20 years (5)	2	3	33	7		0	0	13	6		0	0	20	8	
More than 20 years (6)	2	3	20	4		1	4	6	3		3	7	14	6	
Unknown	5	8	5	1		1	4	4	2		0	0	0	0	
**Sour Taste Present**															
No (0)	32	49	172	34	0.014	16	59	74	35	0.005	20	48	80	32	0.067
Yes (1)	25	38	279	56		6	22	117	55		22	52	172	68	
Unknown	8	12	50	10		5	19	23	11		0	0	0	0	
**Sour Taste Frequency**															
Never (0)	39	60	226	45	0.091	20	74	99	46	0.028	20	48	81	32	0.538
Annually or less (1)	5	8	23	5	FET	2	7	6	3	FET	0	0	4	2	FET
Few times a year (2)	3	5	48	10		2	7	11	5		4	10	40	16	
Few times a month (3)	5	8	47	9		1	4	26	12		6	14	38	15	
Few times a week (4)	9	14	78	16		1	4	37	17		7	17	45	18	
Daily (5)	3	5	67	13		1	4	29	14		5	12	44	17	
Unknown	1	2	12	2		0	0	6	3		0	0	0	0	
**Frequency of Symptoms Preventing Eating and Drinking**															
Never (0)	24	37	209	42	0.003	13	48	102	48	0.039	9	21	83	33	0.029
Few times a year (1)	3	19	25	5	FET	1	4	3	1	FET	4	10	30	12	FET
Few times a month (2)	5	8	110	22		2	7	50	23		1	2	34	13	
Few times a week (3)	5	8	69	14		1	4	30	14		9	21	33	13	
Daily (4)	21	32	82	16		7	26	26	12		19	45	72	29	
Unknown	7	11	6	1		3	11	3	1		0	0	0	0	
**Swallowing Difficulty Present**															
No (0)	19	29	256	51	0.002	10	37	109	51	0.398	18	43	154	61	0.040
Yes (1)	44	68	242	48		15	56	104	49		24	57	98	39	
Unknown	2	3	3	1		2	7	1	0		0	0	0	0	
**Dysphagia Score**															
No dysphagia (0)	19	29	256	51	0.003	10	37	109	51	0.486	18.0	43	155	62	0.538
Dysphagia to solids (1)	19	29	120	24		8	30	47	22	FET	17	40	61	24	FET
Dysphagia to solids and semi-solids (2)	14	22	49	10		5	19	28	13		1	2	15	6	
Dysphagia to solids, semi-solids and liquids (3)	11	17	64	13		2	7	23	11		6	14	20	8	
Unknown	2	3	12	2		2	7	7	3		0	0	1	0	
**Swallowing Pain Present**															
No (0)	33	51	317	63	0.003	17	63	138	64	0.455	27	64	222	88	<0.001
Yes (1)	27	42	110	22		8	30	41	19		15	36	30	12	
Unknown	5	8	74	15		2	7	35	16			0	0	0	
**Unexplained Weight Loss Present**															
No (0)	28	36	369	74	<0.001	17	63	148	69	0.466	18	43	200	79	<0.001
Yes (1)	36	55	128	26		10	37	64	30		24	57	52	21	
Unknown	1	2	4	1		0	0	2	1		0	0	0	0	
**Weight Loss (kg)**															
Mean	5.9		1.8		<0.001	2.6		2.5		0.033	6.0		1.5		<0.001
SD	7.8		4.6		KW	4.6		5.5		KW	7.0		3.6		KW
Unknown	3		36			1		12			21		33		
**Fruit and Vegetable Frequency**															
Rarely or never (0)	3	5	8	2	0.003	0	0	8	4	0.516	8	19	11	4	0.538
Few times each month (1)	6	9	14	3	FET	0	0	2	1	FET	0	0	9	4	FET
Few times each week (2)	18	28	114	23		7	26	45	21		6	14	53	21	
Few times each day (3)	27	42	198	40		13	48	85	40		18	43	109	43	
5 a day or more (4)	10	15	162	32		5	19	71	33		10	24	70	28	
Unknown	1	2	5	1		2	7	3	1		0	0	0	0	
**Known Psychological Disorders**															
No (0)	52	80	324	65	0.002	24	89	137	64	0.008	32	76	147	58	0.043
Yes (1)	7	11	153	31		1	4	63	29		10	24	105	42	
Unknown	6	9	24	5		2	7	14	7		0	0	0	0	
**Butterflies Feeling**															
Not at all (0)	25	38	153	31	0.362	15	56	80	37	0.257	26	62	107	42	0.076
Occasionally (1)	26	40	219	44	FET	8	30	81	38	FET	7	17	82	33	FET
Quite often (2)	4	6	47	9		0	0	13	6		2	5	24	10	
Very often (3)	0	0	14	3		0	0	7	3		3	7	23	9	
Unknown	10	15	68	14		4	15	33	15		4	10	16	6	
**Read Local Newspaper**															
No, never (0)	16	25	101	20	0.048	6	22	41	19	0.888	16	38	131	52	0.270
Yes, rarely (1)	17	26	86	17		5	19	46	21		7	17	31	12	
Yes, sometimes (2)	10	15	133	27		5	19	54	25		8	19	49	19	
Yes, often (3)	14	22	157	31		9	33	68	32		11	26	41	16	
Unknown	8	12	24	5		2	7	5	2		0	0	0	0	

A total of 807 patients were included in model training (566 patients) and testing (241 patients), while 294 patients were included in model validation. Full demographic information and breakdown is presented in Table 1. A total of 65, 27 and 42 oesophageal and gastro oesophageal junction (GOJ) cancer cases were included in the training, testing and validation datasets respectively. TNM staging information, where available, is presented in Supplementary Table 2. 80%, 93% and 83% of cancers in the training, testing and validation datasets respectively were OACs. There was no statistically significant difference in the distribution on either the cancer site (Χ^2^ = 2.21, *p* = 0.33) and or the histology (Χ^2^ = 5.06, *p* = 0.28) within the three datasets. Notably, cancer patients were older and more likely to have had some quantified weight loss across the three datasets. Cancer patients across all three datasets were less likely to suffer from psychological disorders compared to non-cancer patients.

### Selection of features

251 features were available for analysis, which includes data from the questionnaire, endoscopy and histology results. Prior to imputation we removed 101 features which had data missing in more than 20% of patients. We subsequently selected 17 features which were top ranked for the prediction of oesophageal and GOJ cancers (Supplementary Table 3). Top ranked features were multidimensional and included demographic information, symptoms and psychological and food related variables. These top ranked features were selected for ML model development.

### Development of ML model

**Fig. 2:**
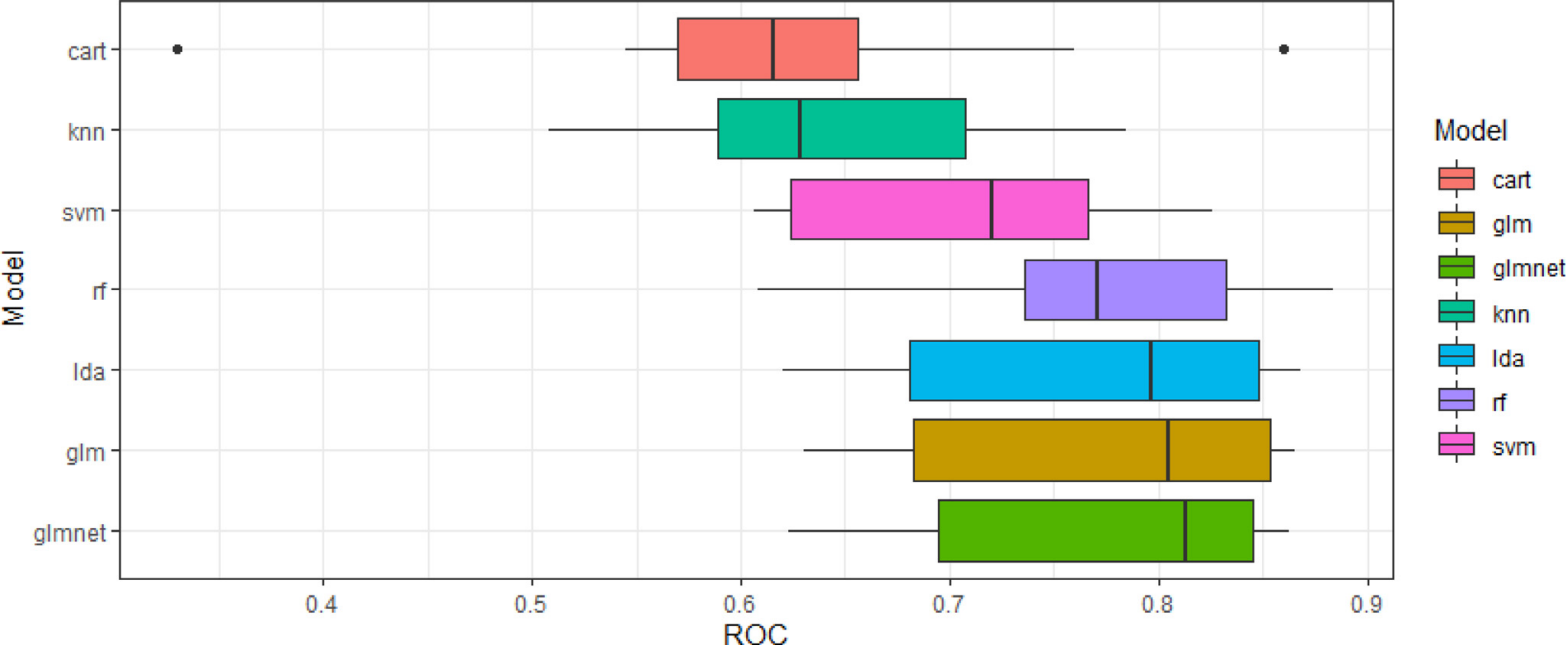
Boxplot of each model and their spread of area under the receiver operating characteristic curve (ROC) for each model. Table demonstrates median ROC, sensitivity and specificity and their respective inter-quartile range (IQR) during model development using 10-fold cross-validation.

Fig. 2 demonstrates the distribution of area under the receiver operating characteristic curve (AUC) after 10-fold cross-validation in the training dataset, as well as median and IQR for AUC, sensitivity and specificity for the seven different ML methods. The best performing model with the highest median AUC was regularised logistic regression (AUC: 0.81, IQR: 0.69–0.85). The regularised logistic regression model was associated with an alpha value of 0.1 and a lambda value of 0.0149. We selected this model for testing and validation and for determining appropriate cut offs in the cost function.

### Testing and validation of model

**Fig. 3:**
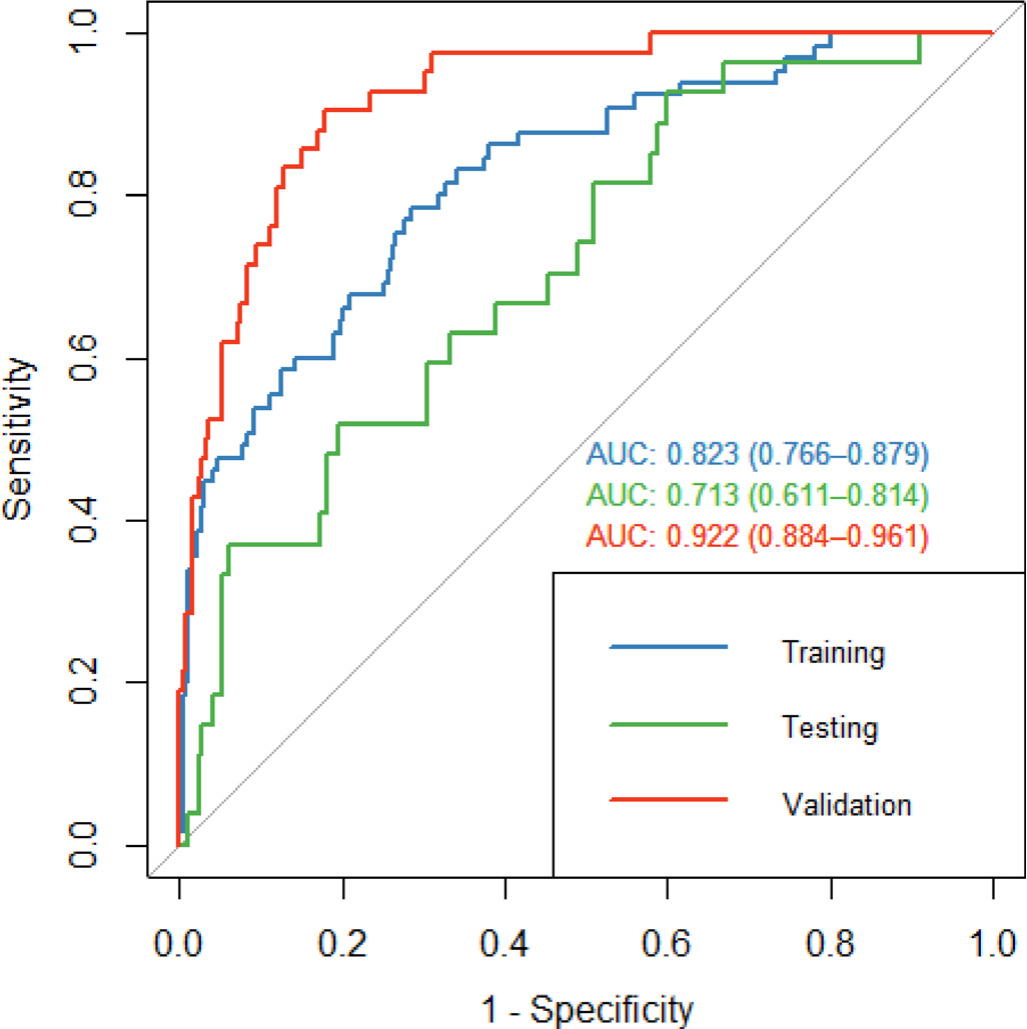
ROC curve for training, testing and validation datasets for regularised logistic regression for predicting oesophageal and GOJ cancer.

Fig. 3 demonstrates the receiver operating characteristic (ROC) curve for regularised logistic regression, with the final model reapplied to the training dataset for reference purposes and tested on both the testing and independent validation datasets. For the testing data set the model achieved an AUC of 0.71 (95% CI: 0.61–0.81). For validation, the model achieved an AUC of 0.92 (95% CI: 0.88–0.96). Table 2 demonstrates the sensitivity, specificity, positive predictive value (PPV) and negative predictive value (NPV) for each dataset with the cost function applied. Our final model achieved a sensitivity and specificity of 96.3% and 20.1% for the testing dataset. For the validation dataset, the sensitivity and specificity were 97.6% and 59.1% respectively. The final model with coefficients is presented in Supplementary Table 4.

**Table 2:** Sensitivity, specificity, positive predictive value (PPV) and negative predictive value (NPV) with 95% confidence intervals (95% CI) for regularised logistic regression. This was set at a cut off of 0.03 (false negatives are weighted 50x more than false positive cases).

Dataset	Sensitivity (95% CI) (%)	Specificity (95% CI) (%)	Positive Predictive Value (95% CI) (%)	Negative Predictive Value (95% CI) (%)
Training Data	96.9 (89.3–99.6)	24.4 (20.7–28.4)	14.3 (11.1–17.9)	98.4 (94.3–99.8)
Testing Data	96.3 (81.0- 99.9)	20.1 (14.9–26.1)	13.2 (8.8–18.7)	97.7 (88.0–99.9)
Validation Data	97.6 (87.4–99.9)	59.1 (52.8–65.3)	28.5 (21.3–36.6)	99.3 (96.3–100.0)
Validation Data (Gastric Cancer Cases)	75.0 (42.8–94.5)	59.1(52.8–65.3)	8.0 (3.7–14.7)	98.0 (94.3–99.6)

### Performance of model by histological subtype

We assessed the performance of our model on different histological subtypes of cancer. The model was able to predict correctly all 3 OSCC cases in the testing dataset and all 5 OSCC cases in the validation dataset as cancer. For OAC, all 21 cases were correctly predicted in the testing dataset, while all but one case (38/39; 97.4%) was correctly predicted as cancer in the validation dataset.

### Pilot testing with gastric cancer cases

In a further sub analysis, we assessed if our model was able to predict the presence of gastric cancers. Demographic information for this subgroup of patient is presented in Supplementary Table 5 and includes 12 gastric cancer cases with the same controls as the original validation dataset. Supplementary Figure 1 demonstrates the ROC curve for validation where it achieved an AUC of 0.78 (95% CI: 0.62–0.95). At the same cut off level, this led to a sensitivity of 75.0% (Table 2).

## Discussion

Using an ML approach, we have created a risk stratification tool which could be used as a diagnostic aid to identify patients who may have oesophageal or gastro-oesophageal junction (GOJ) cancer. Our model is based on demographic factors such as age and sex which are routinely captured, as well as alarm features such as dysphagia and unintentional weight loss which are currently included in National Institute for Health and Care Excellence (NICE) UGI cancer referral guidelines [35]. Our model further incorporates additional features, such as known psychological disorders, which we believe gives an additional richness to the model and helps improve its performance. In particular, we have also demonstrated the robustness of the models by both testing and independently validating the model. We were able to achieve a sensitivity of over 96% for detecting cancer, while specificity ranged from 20% in the testing dataset to 59% in the validation dataset. This large range of results for specificity is likely to be a result of both the relatively small numbers of cancers in both datasets, and also cancer and non-cancer patients were not equally distributed in the datasets. In addition, we also trialled our model on gastric cancer patients. Although the numbers were small it identified 9/12 (75%) gastric cancer patients correctly. It may be possible to enhance accuracy for this condition by capturing symptoms specific to gastric cancer such as early satiety and symptoms of anaemia [36]. Incorporating these features when creating a model may further improve the sensitivity for detecting gastric cancers.

Our work adds to previous research; ML algorithms have previously been developed in a Chinese population to predict the risk of UGI lesions [37]. However, the study incorporated a combined endpoint of UGI pathology. This included both gastric cancer, which had a prevalence of 0.3% in the study population, and more benign pathology such as gastric erosions, which had a prevalence of 22% [37]. Our model differs by emphasising cancers, which we believe will have a greater utility especially in the triaging of suspected cancer 2WW referrals and prioritising procedures which are likely to have a greater yield for serious pathology.

Our model appears to perform well against currently used triaging methods. In our validation dataset we were able to achieve a PPV of 28.5% and an NPV of 99.3%. This improves upon using alarm features solely, which on their own have PPVs ranging from 0 to 11% for oesophageal and gastric cancers, with considerable heterogeneity between studies [38]. Even when using a combination of alarm features, PPVs still remain at around 10% [38]. In addition, our work also improves on the EDS. [12,13]. During the COVID-19 pandemic the EDS has been used for triaging urgent UGI referrals, and a prospective series demonstrated a sensitivity of 96.7% and a specificity of 32.6%, although the authors also included 10 gastric and one duodenal cancer in the outcome [39]. This equated to a PPV of 9.7% and NPV of 99.3% for the detection of UGI cancer [39]. One major advantage of our approach is that it has a higher PPV compared to the EDS, which could be especially advantageous in reducing the number of normal procedures being classed as urgent or being performed. Our model also compares favourably with models recently validated for the detection of incident cases of OAC and GOJ adenocarcinoma. The best model in validation achieved an AUC of 0.73, compared to 0.92 in our study. [40,41].

## Limitations of the study

First, some patients were recruited after the diagnosis of cancer was known. Subsequently, such patients may be subject to recall bias and report higher rates of symptoms. While it would be preferable to perform prospective recruitment, there are challenges associated with this as UGI cancer has a low incidence in the UK [7]. Second, we would have preferred to directly test the performance of the EDS on our cohort of patients, and therefore create head-to-head comparisons. However, we were unable to extrapolate for one of the elements of the score (dysphagia localises to neck) with our existing data. Third, as this study is based in the UK, where OAC is the predominant subtype, our scoring system may be less applicable to other nations or territories where other histological subtypes are more common. Fourth, our model incorporates a large number of features which increases its complexity during clinical use, although with the rise of electronic health record systems, patients are increasingly prepared to complete health questionnaires online. This can rapidly generate rich datasets for the clinician to review easily and is starting to make its way into routine clinical use. Finally, this model needs further validation in larger cohorts to ensure its performance remains consistent and slight adjustment to enhance its performance against gastric cancers. This is especially important to ensure greater consistency in the calculation of model performance metrics.

## Conclusions

Using ML methods, we have created and validated a tool for the prediction of oesophageal and GOJ cancers. This could have real impact in helping to prioritise patients for urgent investigation, particularly at a time of COVID-19 related backlogs.

## Author contributions

KMAH: conceptualisation, data curation, formal analysis, investigation, methodology, software, validation, visualisation, writing -original draft, writing - review & editing

AR: conceptulisation, formal analysis, methology, supervision, writing - review & editing

AH, HM, MD, PW, AW, SC, LH, LM, DGG, VS: investigation, project administration,

SPIT Study Group Collaborators: investigation

AB: conceptualisation, formal analysis, methodology, supervision, writing - review & editing

LBL: conceptualisation, formal analysis, funding acquisition, investigation, methodology, supervision, validation, writing - review & editing

Patient and public involvement: patients and/or the public were not involved in the design, or conduct of this research but were involved in reporting, and dissemination plans.

**Data**: Authors were not precluded from accessing data in the study, and they accept responsibility to submit for publication. KMAH and LBL directly accessed and verified the underlying data. Data is available on application from the senior author (LBL) on reasonable request.

Online Calculator

An online version of our model is available via: https://endopredict.shinyapps.io/endopredict/

## Declaration of Competing Interest

The authors declare that they have no known competing financial interests or personal relationships that could have appeared to influence the work reported in this paper.

## Appendix Supplementary materials

Image, application 1

## Footnotes

Supplementary material associated with this article can be found in the online version at doi:10.1016/j.clinre.2023.102087.

## References

[1] Arnold M. (2020). Global burden of 5 major types of gastrointestinal cancer. Gastroenterology.

[2] Arnold M., Soerjomataram I., Ferlay J., Forman D. (2015). Global incidence of oesophageal cancer by histological subtype in 2012. Gut.

[3] Abnet C.C., Arnold M., Wei W.Q. (2018). Epidemiology of esophageal squamous cell carcinoma. Gastroenterology.

[4] Coleman H.G., Xie S.H., Lagergren J. (2018). The epidemiology of esophageal adenocarcinoma. Gastroenterology.

[5] Cancer Research UK (2018). Cancer research uk.

[6] Bornschein J., Quante M., Jansen M. (2021). The complexity of cancer origins at the gastro-oesophageal junction. Best Pract Res Clin Gastroenterol.

[7] Shawihdi M. (2014). Variation in gastroscopy rate in English general practice and outcome for oesophagogastric cancer: retrospective analysis of Hospital Episode Statistics. Gut.

[8] Ho K.M.A., Banerjee A., Lawler M., Rutter M.D., Lovat L.B. (2021). Predicting endoscopic activity recovery in England after COVID-19: a national analysis. Lancet Gastroenterol Hepatol.

[9] Turkington R.C. (2021). The impact of the COVID-19 pandemic on Barrett's esophagus and esophagogastric cancer. Gastroenterology.

[10] Sud A. (2020). Effect of delays in the 2-week-wait cancer referral pathway during the COVID-19 pandemic on cancer survival in the UK: a modelling study. Lancet Oncol.

[11] Maringe C. (2020). The impact of the COVID-19 pandemic on cancer deaths due to delays in diagnosis in England, UK: a national, population-based, modelling study. Lancet Oncol.

[12] Murray I.A., Palmer J., Waters C., Dalton H.R (2012). Predictive value of symptoms and demographics in diagnosing malignancy or peptic stricture. World J Gastroenterol.

[13] Rhatigan E., Tyrmpas I., Murray G., Plevris J.N (2010). Scoring system to identify patients at high risk of oesophageal cancer. Br J Surg.

[14] Rosenfeld A. (2020). Development and validation of a risk prediction model to diagnose Barrett's oesophagus (MARK-BE): a case-control machine learning approach. Lancet Digit. Heal..

[15] Shung D.L. (2020). Validation of a machine learning model that outperforms clinical risk scoring systems for upper gastrointestinal bleeding. Gastroenterology.

[16] Vakil N., Moayyedi P., Fennerty M.B., Talley N.J. (2006). Limited value of alarm features in the diagnosis of upper gastrointestinal malignancy: systematic review and meta-analysis. Gastroenterology.

[17] Liu X. (2014). Gastro-esophageal reflux disease symptoms and demographic factors as a pre-screening tool for Barrett's esophagus. PLoS One.

[18] Locke G.R., Zinsmeister A.R., Talley N.J (2003). Can symptoms predict endoscopic findings in GERD?. Gastrointest Endosc.

[19] Jones R., Coyne K., Wiklund I. (2007). The Gastro-oesophageal Reflux Disease Impact Scale: a patient management tool for primary care. Aliment Pharmacol Ther.

[20] Steptoe A., Shankar A., Demakakos P., Wardle J. (2013). Social isolation, loneliness, and all-cause mortality in older men and women. Proc Natl Acad Sci USA.

[21] Mykletun A. (2007). Anxiety, depression, and cause-specific mortality: the HUNT study. Psychosom Med.

[22] Friedman G.D., Udaltsova N., Chan J., Quesenberry C.P., Habel L.A. (2009). Screening pharmaceuticals for possible carcinogenic effects: initial positive results for drugs not previously screened. Cancer Causes Control.

[23] Zigmond A.S., Snaith R.P. (1983). The Hospital Anxiety and Depression Scale. Acta Psychiatr Scand.

[24] Hughes M.E., Waite L.J., Hawkley L.C., Cacioppo J.T. (2004). A short scale for measuring loneliness in large surveys: results from two population-based studies. Res Aging.

[25] Mellow M.H., Pinkas H. (1985). Endoscopic laser therapy for malignancies affecting the esophagus and gastroesophageal junction: analysis of technical and functional efficacy. Arch Intern Med.

[26] Ho K.M.A. (2021). PTH-72 A symptom and risk factor questionnaire accurately predicts upper gastrointestinal cancer. Gut.

[27] R. Core Team. R: a language and environment for statistical computing. (2021).

[28] Stekhoven D.J., Bühlmann P. (2012). Missforest-Non-parametric missing value imputation for mixed-type data. Bioinformatics.

[29] Romanski, P., Kotthoff, L. & Schratz, P. FSelector: selecting Attributes. (2021).

[30] Kuhn, M. Caret: classification and regression training. (2021).

[31] Friedman J., Hastie T., Tibshirani R (2010). Regularization paths for generalized linear models via coordinate descent. J. Stat. Softw..

[32] Robin X. (2011). pROC: an open-source package for R and S+ to analyze and compare ROC curves. BMC Bioinf.

[33] Sing T., Sander O., Beerenwinkel N., Lengauer T. (2005). ROCR: visualizing classifier performance in R. Bioinformatics.

[34] Collins G.S., Reitsma J.B., Altman D.G., Moons K.G. (2015). Transparent reporting of a multivariable prediction model for individual prognosis or diagnosis (TRIPOD): the TRIPOD Statement. BMC Med.

[35] National Institute for Health and Care Excellence (2015).

[36] Smyth E.C., Nilsson M., Grabsch H.I., van Grieken N.C., Lordick F. (2020). Gastric cancer. Lancet (London, England).

[37] Liu Y. (2021). Using machine-learning algorithms to identify patients at high risk of upper gastrointestinal lesions for endoscopy. J Gastroenterol Hepatol.

[38] Stapley S. (2013). The risk of oesophago-gastric cancer in symptomatic patients in primary care: a large case-control study using electronic records. Br J Cancer.

[39] Kamran U. (2022). Assessment of the role of the Edinburgh dysphagia score in referral triage in a national service evaluation of the urgent suspected upper gastrointestinal cancer pathway. Aliment Pharmacol Ther.

[40] Rubenstein J.H. (2021). Validation of Tools for Predicting Incident Adenocarcinoma of the Esophagus or Esophagogastric Junction. Am J Gastroenterol.

[41] Kunzmann A.T. (2018). Model for Identifying Individuals at Risk for Esophageal Adenocarcinoma. Clin Gastroenterol Hepatol.

