# Supplementary material for: Development and validation of a multivariable risk factor questionnaire to detect oesophageal cancer in 2-week wait patients
Source: Clin Res Hepatol Gastroenterol. 2023 Mar;47(3):None. doi: 10.1016/j.clinre.2023.102087 (PMC10602932; doi:10.1016/j.clinre.2023.102087)
Supplement: Supplementary file 1 [file mmc1.docx]

## Supplementary Tables

| **Question Group** | **Number of Questions (SPIT)** | **Number of Questions (RISQ)** |
| --- | --- | --- |
| Personal Questions | 8 | 2 |
| Dental Health | 9 |  |
| Heartburn/Acid Reflux | 55 | 4 |
| Dysphagia | 8 | 3 |
| Unexplained Weight Loss | 4 | 2 |
| Nausea and Vomiting | 6 |  |
| Smoking History | 14 | 1 |
| Alcohol | 9 |  |
| Obesity | 4 |  |
| Diet | 19 | 1 |
| Physical Exercise | 6 |  |
| Family History | 12 | 1 |
| Mental Wellbeing | 14 | 1 |
| Medical History | 26 | 1 |
| General Questions | 15 | 1 |

Supplementary Table 1: Breakdown of questions in each group for SPIT and RISQ questionnaires

|  | **Training Data**  **(n=65)** | | **Testing Data**  **(n=27)** | | **Validation Data**  **(n=42)** | |
| --- | --- | --- | --- | --- | --- | --- |
|  | **n** | **%** | **n** | **%** | **n** | **%** |
| **T Staging** | | | | | | |
| T1 | 7 | 11 | 2 | 7.4 | 2 | 4.8 |
| T2 | 4 | 6.2 | 0 | 0 | 1 | 2.4 |
| T3 | 10 | 15 | 3 | 11 | 6 | 14 |
| T4 | 3 | 4.6 | 1 | 3.7 | 4 | 9.5 |
| Unknown | 41 | 63 | 21 | 78 | 29 | 69 |
| **N Staging** | | | | | | |
| N0 | 8 | 12 | 2 | 7.4 | 7 | 17 |
| N1 | 9 | 14 | 3 | 11 | 3 | 7.1 |
| N2 | 3 | 4.6 | 1 | 3.7 | 3 | 7.1 |
| N3 | 2 | 3.1 | 0 | 0 | 0 | 0 |
| Unknown | 43 | 66 | 21 | 78 | 29 | 69 |
| **M Staging** | | | | | | |
| M0 | 16 | 25 | 4 | 15 | 11 | 26 |
| M1 | 4 | 6.2 | 1 | 3.7 | 2 | 4.8 |
| Unknown | 45 | 69 | 22 | 82 | 29 | 69 |

Supplementary Table 2: TNM staging information for oesophageal and GOJ cancer cases

| **Feature** | **Information Gain** | **Correlation Based Feature Selection** | **Overall**  **Rank** |
| --- | --- | --- | --- |
| Frequency of Symptoms Preventing Eating and Drinking | 0.015 | 0.175 | 1= |
| Unexplained Weight Loss (kg) | 0.014 | 0.184 | 1= |
| Age | 0.016 | 0.167 | 3 |
| Unexplained Weight Loss Present | 0.013 | 0.167 | 4 |
| Known Psychological Disorders | 0.013 | 0.149 | 5 |
| Sour Taste Frequency | 0.012 | 0.152 | 6 |
| Chest Pain Begin | 0.012 | 0.147 | 7= |
| Sex | 0.011 | 0.148 | 7= |
| Fruit and Vegetable Frequency | 0.009 | 0.136 | 9 |
| Regurgitation Begin | 0.008 | 0.127 | 10 |
| Sour Taste Present | 0.007 | 0.122 | 11= |
| Swallowing Pain Present | 0.007 | 0.125 | 11= |
| Read Local Newspaper | 0.006 | 0.113 | 13= |
| Dysphagia Score | 0.006 | 0.116 | 13= |
| Butterflies Feeling | 0.006 | 0.106 | 15 |
| Swallowing Difficulty Present | 0.005 | 0.096 | 16 |
| Current/Previous Smoking | 0.003 | 0.073 | 17 |

Supplementary Table 3: Ranking of information gain and correlation based feature selection to produce a final model.

| **Feature** | **Coefficient** |
| --- | --- |
| *Intercept* | *-2.539* |
| Age | 0.440 |
| Female Sex | -0.416 |
| Chest Pain Begin | -0.036 |
| Regurgitation Begin | -0.047 |
| Sour Taste Present | (feature dropped) |
| Sour Taste Frequency | -0.223 |
| Frequency of Symptoms Preventing Eating and Drinking | 0.135 |
| Swallowing Difficulty Present | 0.155 |
| Dysphagia Score | 0.187 |
| Swallowing Pain Present | 0.222 |
| Unexplained Weight Loss Present | 0.263 |
| Unexplained Weight Loss (kg) | 0.183 |
| Current/Previous Smoking | 0.087 |
| Fruit and Vegetable Frequency | -0.228 |
| Known Psychological Disorders | -0.335 |
| Butterflies Feeling | -0.151 |
| Read Local Newspaper | -0.282 |

Supplementary Table 4: Final model from regularised logistic regression where alpha=0.1 and lambda=0.0149. A web-based calculator is available from: <https://endopredict.shinyapps.io/endopredict/>

|  | **Pilot Data (Gastric) (n=294)** | | | | |
| --- | --- | --- | --- | --- | --- |
|  | **Cancer Present** | **%** | **Cancer Absent** | **%** | **p value** |
| **n** | 12 | 5 | 252 | 95 |  |
| **Age** |  |  |  |  |  |
| Mean | 63.2 |  | 52.1 |  | <0.001 |
| SD | 15.5 |  | 17.1 |  |  |
| **Sex** |  |  |  |  |  |
| Male (1) | 35 | 83 | 137 | 54 | <0.001 |
| Female (2) | 7 | 17 | 115 | 46 |  |
| **Cancer Diagnosis** |  |  |  |  |  |
| Oesophageal | 0 | 0 |  |  |  |
| Oesophago-gastric | 0 | 0 |  |  |  |
| Gastric | 12 | 100 |  |  |  |
| **Cancer Histology** |  |  |  |  |  |
| Adenocarcinoma | 12 | 100 |  |  |  |
| Squamous Cell Carcinoma | 0 | 0 |  |  |  |
| Other/Unknown | 0 | 0 |  |  |  |
| **Ethnicity** |  |  |  |  |  |
| White British/Irish/European | 9 | 75 | 190 | 75 | 0.819 |
| Mixed Race | 0 | 0 | 5 | 2 | FET |
| Asian | 0 | 0 | 10 | 4 |  |
| Asian Other | 0 | 0 | 13 | 5 |  |
| Black | 0 | 0 | 12 | 5 |  |
| Other | 3 | 25 | 22 | 9 |  |
| **Current/Previous Smoking** |  |  |  |  |  |
| No (0) | 4 | 33 | 137 | 54 | 0.258 |
| Yes (1) | 8 | 67 | 115 | 46 |  |
| Unknown | 0 | 0 | 0 | 0 |  |
| **Smoking Pack Years** |  |  |  |  |  |
| Mean | 3.4 |  | 7.1 |  | 0.984 |
| SD | 6.4 |  | 15.0 |  | KW |
| Unknown | 4 |  | 14 |  |  |
| **BMI** |  |  |  |  |  |
| Mean | 23.5 |  | 30.9 |  | 0.165 |
| SD | 5.8 |  | 10.2 |  |  |
| Unknown | 9 |  | 223 |  |  |
| **Chest Pain Begin** |  |  |  |  |  |
| No Chest Pain (0) | 12 | 100 | 123 | 49 | 0.182 |
| Less than 6 months (1) | 0 | 0 | 13 | 5 | FET |
| 6 months to 1 year (2) | 0 | 0 | 18 | 7 |  |
| 1 to 5 years (3) | 0 | 0 | 37 | 15 |  |
| 5 to 10 years (4) | 0 | 0 | 24 | 10 |  |
| 10 to 20 years (5) | 0 | 0 | 23 | 9 |  |
| More than 20 years (6) | 0 | 0 | 14 | 6 |  |
| Unknown | 0 | 0 | 0 | 0 |  |
| **Regurgitation Begin** |  |  |  |  |  |
| No Regurgitation (0) | 9 | 75 | 122 | 48 | 0.726 |
| Less than 6 months (1) | 1 | 8 | 11 | 4 | FET |
| 6 months to 1 year (2) | 0 | 0 | 19 | 8 |  |
| 1 to 5 years (3) | 1 | 8 | 40 | 16 |  |
| 5 to 10 years (4) | 1 | 8 | 26 | 10 |  |
| 10 to 20 years (5) | 0 | 0 | 20 | 8 |  |
| More than 20 years (6) | 0 | 0 | 14 | 6 |  |
| Unknown | 0 | 0 | 0 | 0 |  |
| **Sour Taste Present** |  |  |  |  |  |
| No (0) | 4 | 33 | 80 | 32 | 1.000 |
| Yes (1) | 8 | 67 | 172 | 68 | FET |
| Unknown | 0 | 0 | 0 | 0 |  |
| **Sour Taste Frequency** |  |  |  |  |  |
| No (0) | 4 | 33 | 81 | 32 | 0.797 |
| Annually or less (1) | 0 | 0 | 4 | 2 | FET |
| Few times a year (2) | 1 | 8 | 40 | 16 |  |
| Few times a month (3) | 3 | 25 | 38 | 15 |  |
| Few times a week (4) | 3 | 25 | 45 | 18 |  |
| Daily (5) | 1 | 8 | 44 | 17 |  |
| Unknown | 0 | 0 | 0 | 0 |  |
| **Frequency of Symptoms Preventing Eating and Drinking** | | | |  |  |
| Never (0) | 6 | 50 | 83 | 33 | 0.554 |
| Few times a year (1) | 0 | 0 | 30 | 12 | FET |
| Few times a month (2) | 2 | 17 | 34 | 13 |  |
| Few times a week (3) | 2 | 17 | 33 | 13 |  |
| Daily (4) | 2 | 17 | 72 | 29 |  |
| Unknown | 0 | 0 | 0 | 0 |  |
| **Swallowing Difficulty Present** |  |  |  |  |  |
| No (0) | 11 | 92 | 154 | 61 | 0.035 |
| Yes (1) | 1 | 8 | 98 | 39 | FET |
| Unknown | 0 | 0 | 0 | 0 |  |
| **Dysphagia Score** |  |  |  |  |  |
| No dysphagia (0) | 11.0 | 92 | 155 | 62 | 0.797 |
| Dysphagia to solids (1) | 1 | 8 | 61 | 24 | FET |
| Dysphagia to solids and semi-solids (2) | 0 | 0 | 15 | 6 |  |
| Dysphagia to solids, semi-solids and liquids (3) | 0 | 0 | 20 | 8 |  |
| Unknown | 0 | 0 | 1 | 0 |  |
| **Swallowing Pain Present** |  |  |  |  |  |
| No (0) | 12 | 100 | 222 | 88 | 0.371 |
| Yes (1) | 0 | 0 | 30 | 12 | FET |
| Unknown | 0 | 0 | 0 | 0 |  |
| **Unexplained Weight Loss Present** |  |  |  |  |  |
| No (0) | 3 | 25 | 200 | 79 | <0.001 |
| Yes (1) | 9 | 75 | 52 | 21 | FET |
| Unknown | 0 | 0 | 0 | 0 |  |
| **Weight Loss (kg)** |  |  |  |  |  |
| Mean | 9.0 |  | 1.5 |  | <0.001 |
| SD | 7.0 |  | 3.6 |  | KW |
| Unknown | 6 |  | 33 |  |  |
| **Fruit and Vegetable Frequency** |  |  |  |  |  |
| Rarely or never (0) | 2 | 17 | 11 | 4 | 0.797 |
| Few times each month (1) | 0 | 0 | 9 | 4 | FET |
| Few times each week (2) | 3 | 25 | 53 | 21 |  |
| Few times each day (3) | 5 | 42 | 109 | 43 |  |
| 5 a day or more (4) | 2 | 17 | 70 | 28 |  |
| Unknown | 0 | 0 | 0 | 0 |  |
| **Known Psychological Disorders** |  |  |  |  |  |
| No (0) | 9 | 75 | 147 | 58 | 0.370 |
| Yes (1) | 3 | 25 | 105 | 42 | FET |
| Unknown | 0 | 0 | 0 | 0 |  |
| **Butterflies Feeling** |  |  |  |  |  |
| Not at all (0) | 6 | 50 | 107 | 42 | 0.843 |
| Occasionally (1) | 3 | 25 | 82 | 33 | FET |
| Quite often (2) | 1 | 8 | 24 | 10 |  |
| Very often (3) | 0 | 0 | 23 | 9 |  |
| Unknown | 2 | 17 | 16 | 6 |  |
| **Read Local Newspaper** |  |  |  |  |  |
| No, never (0) | 5 | 42 | 131 | 52 | 0.671 |
| Yes, rarely (1) | 1 | 8 | 31 | 12 | FET |
| Yes, sometimes (2) | 4 | 33 | 49 | 19 |  |
| Yes, often (3) | 2 | 17 | 41 | 16 |  |
| Unknown | 0 | 0 | 0 | 0 |  |

**Supplementary Table 5: Demographic table for patients diagnosed with gastric cancer included in pilot validation. Numbers in brackets following feature (e.g. no, never (0)) denote coding used for model development. p-values are for chi-squared tests unless otherwise stated. FET= Fisher's Exact Test. KW=Kruskal Wallis Test**

## Supplementary Figures


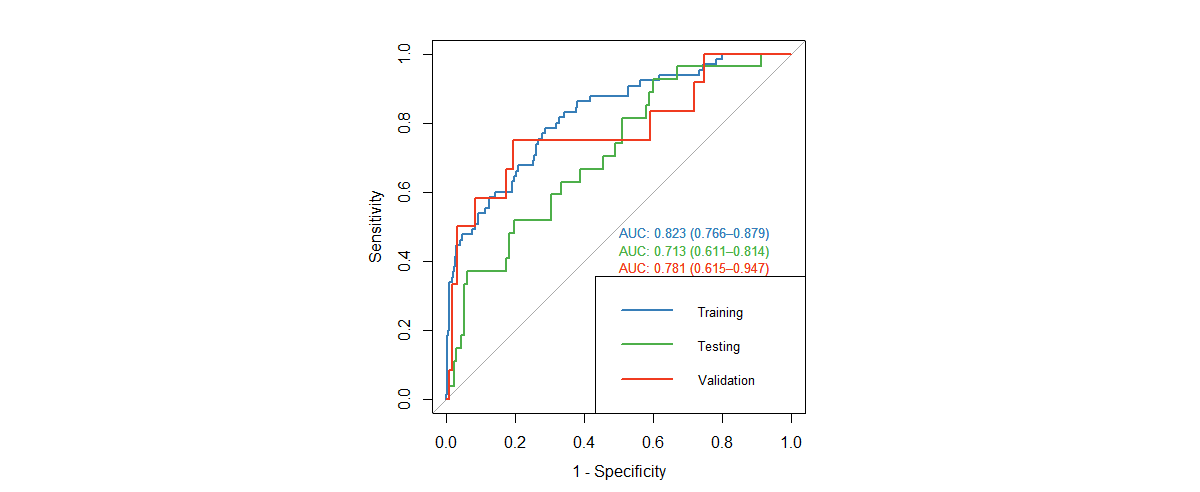


Supplementary Figure 1: ROC curve for training, testing and validation datasets for regularised logistic regression for predicting gastric cancer
